# Stress granule activation attenuates lipopolysaccharide-induced cardiomyocyte dysfunction

**DOI:** 10.1186/s12872-023-03281-0

**Published:** 2023-05-27

**Authors:** Yaqiao Wang, Runmin Liu, Kehan Wu, Gaowei Yang, Yusheng Wang, Hao Wang, Tao Rui

**Affiliations:** Division of Cardiology, Department of Medicine, the Affiliated People’s Hospital of Jiangsu University, Zhenjiang, Jiangsu China

**Keywords:** Stress granule, Sepsis, Myocardial dysfunction, G3BP1, Mitochondria

## Abstract

**Background:**

Sepsis is the leading cause of death in intensive care units. Sepsis-induced myocardial dysfunction, one of the most serious complications of sepsis, is associated with higher mortality rates. As the pathogenesis of sepsis-induced cardiomyopathy has not been fully elucidated, there is no specific therapeutic approach. Stress granules (SG) are cytoplasmic membrane-less compartments that form in response to cellular stress and play important roles in various cell signaling pathways. The role of SG in sepsis-induced myocardial dysfunction has not been determined. Therefore, this study aimed to determine the effects of SG activation in septic cardiomyocytes (CMs).

**Methods:**

Neonatal CMs were treated with lipopolysaccharide (LPS). SG activation was visualized by immunofluorescence staining to detect the co-localization of GTPase-activating protein SH3 domain binding protein 1 (G3BP1) and T cell-restricted intracellular antigen 1 (TIA-1). Eukaryotic translation initiation factor alpha (eIF2α) phosphorylation, an indicator of SG formation, was assessed by western blotting. Tumor necrosis factor alpha (TNF-α) production was assessed by PCR and enzyme-linked immunosorbent assays. CMs function was evaluated by intracellular cyclic adenosine monophosphate (cAMP) levels in response to dobutamine. Pharmacological inhibition (ISRIB), a G3BP1 CRISPR activation plasmid, and a G3BP1 KO plasmid were employed to modulate SG activation. The fluorescence intensity of JC-1 was used to evaluate mitochondrial membrane potential.

**Results:**

LPS challenge in CMs induced SG activation and resulted in eIF2α phosphorylation, increased TNF-α production, and decreased intracellular cAMP in response to dobutamine. The pharmacological inhibition of SG (ISRIB) increased TNF-α expression and decreased intracellular cAMP levels in CMs treated with LPS. The overexpression of *G3BP1* increased SG activation, attenuated the LPS-induced increase in TNF-α expression, and improved CMs contractility (as evidenced by increased intracellular cAMP). Furthermore, SG prevented LPS-induced mitochondrial membrane potential dissipation in CMs.

**Conclusion:**

SG formation plays a protective role in CMs function in sepsis and is a candidate therapeutic target.

**Supplementary Information:**

The online version contains supplementary material available at 10.1186/s12872-023-03281-0.

## Background

Sepsis is a life-threatening organ dysfunction caused by a dysregulated host response to infection [1]. It is associated with high morbidity and mortality rates among patients in the intensive care unit [2]. Sepsis-induced myocardial dysfunction (SIMD) is the most common and serious organ dysfunction [3]. Myocardial dysfunction in patients with sepsis is associated with multiple organ failure and poor outcomes [4]. Given that the mechanism underlying septic cardiomyopathy has not been fully elucidated, effective treatment approaches are lacking [5]. Therefore, it is imperative to explore new pathways and therapeutic targets for SIMD.

Stress granules (SG) are stress-induced membrane-less compartments formed in response to various stresses, such as oxidative stress, heat shock, ultraviolet light injury, and viral infection [6–8]. To reduce mRNA mismatch under pressure or save energy, eukaryotic cells form SG to inhibit translation by sorting and “housing” mRNAs, thereby affecting cell metabolism and survival [9, 10]. SG activation can be divided into an eIF2a-phosphorylation pathway and eIF2a-phosphorylation-independent pathway. The SG composition differs among cell types and stress conditions. Generally, the core proteins of the SG include G3BP1 and TIA-1; other major components include mRNAs, subunits of the eIF3, eIF4A, and eIF4G complex, and various RNA-binding proteins. SG activation is driven by liquid-liquid separation. SG can rapidly exchange components with the peripheral cytoplasm or adjacent organelles (such as P-bodies). Once the pressure is relieved, SG disassemble by autophagy, followed by translational recovery.

As a dynamic regulatory platform, SG activation indicates an “emergency state” and affects many diseases via various signaling pathways, especially diseases involving neurodegeneration [11–13]. The functions of SG in diseases are controversial [11, 14]. Previous studies have demonstrated that SG contribute to multiple neurodegenerative diseases, such as amyotrophic lateral sclerosis, Parkinson’s disease, and Alzheimer’s disease [15, 16], while others have reported that SG exert a protective effect, enabling cells to survive under stress conditions [17, 18]. However, the role of SG in SIMD is unknown.

Here, we demonstrated that SG have a cardioprotective role in SIMD. First, we proved that SG are activated in LPS-challenged cardiomyocytes (CMs) by immunofluorescence staining. Then, we evaluated the effects of SG inhibition and activation on the LPS-induced inflammatory response and myocardial dysfunction. In addition, we found that the effect of SG on myocardial function is mediated by interactions with mitochondrial function.

## Methods

### Antibodies and reagents

The *G3BP1* CRISPR/Cas9 KO Plasmid (m) (sc-424175), *G3BP1* CRISPR Activation Plasmid (m) (sc-424175-ACT), Transfection Reagent (sc-395739), Plasmid Transfection Medium (sc-108062), and anti-TIA1(G-3) (sc-166247) and anti-β-actin (sc-8432) antibodies were obtained from Santa Cruz Biotechnology (Dallas, TX, USA). The cAMP Direct Immunoassay Kit (Colorimetric) (K371) was purchased from BioVision (Milpitas, CA, USA). The TNF-α ELISA Kit (E-EL-R2856c) was procured from Elabscience (Wuhan, China). Anti-p-eIF2α (Ser51) (#3597) and anti- eIF2α (#5324) antibodies were purchased from Cell Signaling Technology (Danvers, MA, USA). Anti-G3BP1 (#PA5-90073) antibody was bought from Invitrogen (Waltham, MA, USA). The SG pharmacological inhibitor ISRIB and DAPI (5942) were procured from Sigma-Aldrich (St. Louis, MO, USA). Horseradish peroxidase (HRP)-conjugated goat anti-mouse IgG (115-035-003) and goat anti-rabbit IgG (111-035-003) secondary antibodies were obtained from Jackson ImmunoResearch (West Grove, PA, USA). TRIzol reagent was from Thermo Fisher Scientific (Santa Clara, CA, USA). The RNA Reverse Transcription Kit and qPCR Kit were procured from Vazyme (Nanjing, China). Liberase was purchased from Roche (Shanghai, China). Dulbecco’s Modified Eagle Medium (DMEM) (319-006-CL), fetal bovine serum (FBS, 086–150), and antibiotic-antimycotic (450-115-EL) were purchased from Wisent (QC, Canada). The Mitochondrial Membrane Potential Assay Kit with JC-1 (C2006), Enhanced BCA Protein Assay Kit, Protease and Phosphatase Inhibitor (P1046), and phenylmethanesulfonyl fluoride (PMSF, ST506) were obtained from Beyotime (Shanghai, China). Immobilon ECL Western HRP Substrate (WBKLS0500) and Immobilon PVDF membranes (IPVH00010) were procured from Merck KGaA (Darmstadt, Germany).

### Preparation of neonatal cardiomyocytes

The experimental procedures were conducted under AVMA Guidelines for the Euthanasia of Animals and were approved by the Institutional Animal Care and Use Committee of Jiangsu University (No. UJS-IACUC-2,020,052,001). Newborn wild-type mice (C57BL6) were purchased from the Animal Center of Jiangsu University. Neonatal CMs were isolated and cultured as described previously [19] with some modifications. Briefly, newborn mice (24 h after birth) were euthanized by cervical dislocation, and their hearts were removed aseptically and immersed in cold Ca^2+^- and Mg^2+^-free Hanks’ balanced salt solution (HBSS). The tissue was minced, transferred into Ca^2+^- and Mg^2+^-free HBSS containing 10 µg/mL Liberase, and allowed to dissociate for 15 min at 37 °C. This digestion procedure was repeated 5–6 times. The cells were resuspended in DMEM supplemented with 10% FBS. The CMs were enriched with a pre-plating approach to avoid contamination in Petri dishes for 1 h with 5% CO_2_ at 37 °C and 95% humidity. Then, the myocyte-rich supernatants were centrifuged for 5 min at 250 × *g.* The pellets were resuspended in DMEM supplemented with antibiotic-antimycotic and 10% FBS 100 U/mL penicillin G, and 100 mg/mL streptomycin and seeded into 24-well tissue culture plates (Costar). After 48 h of culture with 5% CO_2_ at 37 °C and 95% humidity, the cells formed a confluent monolayer consisting of 95% myocytes beating in synchrony and were used in experiments.

### In vitro model of sepsis

CMs were exposed to 10 µg/mL LPS in DMEM to establish an in vitro model of sepsis (endotoxemia). In the control group, cells were administered DMEM with an equal volume of saline.

### G3BP1 CRISPR plasmid transfection

The cytoskeleton regulates SG formation, and *G3BP1* is a cytoskeletal protein that provides binding sites for SG activation [20]. *G3BP1* is enriched in intrinsically disordered regions that drive the liquid-liquid phase separation of cytoplasmic contents for SG targeting. Previous studies have shown that the overexpression or knockout of *G3BP1* can increase or reduce SG formation [21]. CMs were transfected with a *G3BP1* CRISPR activation plasmid or *G3BP1* CRISPR/Cas9 KO plasmid according to the manufacturer’s instructions. Briefly, the CMs were seeded in 24-well cell culture plates (5 × 10^5^ cells/well) and cultured for 24 h at 37 °C with 5% CO_2_ and 95% humidity. A mixture of the plasmid and transfection reagent prepared in 150 µL of Plasmid Transfection Medium was added to each well containing cells and incubated for an additional 24 h. Cells were cultured in fresh medium for 48 h for subsequent experiments.

### Western blot analysis

Intracellular p-eIF2α, eIF2α, and β-actin were detected by western blotting. Briefly, CMs were lysed in RIPA lysis buffer (with 20 mM PMSF), and protein concentrations were measured using a BCA Kit. Lysates were separated by SDS-PAGE and transferred onto PVDF membranes. Then, the membranes were incubated with respective primary antibodies overnight at 4 °C. Antibodies against p-eIF2α (Ser51), eIF2α, and β-actin were used. Then, the HRP-conjugated secondary antibody was bound to the PVDF. Finally, the specific protein bands were visualized using ECL.

### Intracellular cyclic adenosine monophosphate (cAMP)

In CMs and myocardial tissue, an increase in intracellular cAMP in response to β-adrenergic agonist stimulation is often associated with enhanced myocardial contractility. An impaired intracellular cAMP response to β-adrenergic drugs has been reported in studies of in vitro sepsis models [22]. Dobutamine (DOB) is a common β-adrenergic receptor agonist. To clarify the change in CMs contractility in the LPS endotoxemia model, an enzyme-linked immunosorbent assay (ELISA) was used to detect the change in intracellular cAMP in response to DOB stimulation as an indicator of CMs contractility. After 8 h of LPS treatment (10 µg/mL), CMs were washed once and incubated for 15 min with DOB (7.5 mM) or normal saline, and then the CMs lysate with hydrochloric acid was used to detect intracellular cAMP using the cAMP Direct Immunoassay Kit.

### Pharmacological inhibition (ISRIB)

ISRIB is an inhibitor of the integrated stress response and inhibits the activation of SG by blocking eIF2α phosphorylation [23]. CMs were pre-treated with ISRIB (200 mM) for 30 min and then co-treated with LPS (10 µg/mL) and ISRIB (200 nM) to inhibit SG formation.

### Enzyme-linked immunosorbent assay

TNF-α and cAMP levels in cell culture supernatants or intracellular homogenates were detected using an ELISA Kit. A 50 µL sample was used following the manufacturer’s instructions. All standards and specimens were measured in duplicate. The data are expressed as pg/mL.

### RNA extraction and RT-qPCR

Total RNA was extracted from cells using TRIzol reagent. The RNA Reverse Transcription Kit was used for reverse transcription. ChamQ Universal SYBR qPCR Master Mix was used for real-time RT-qPCR. Primers were designed by Shanghai Sangon Biotech as follows: *TNF-α*: 5′-CTTCTCATTCCTGCTCGTG-3′ (forward), 5′- TTTGGGAACTTCTCCTCCT-3′ (reverse); and *β-actin*, 5′-TGTCACCAAC TGGGACGATA-3′ (forward), 5′-GGGGTGTTGAAGGTCTCAAA-3′ (reverse). The housekeeping gene *β-actin* was used as a reference to calculate the relative expression of the target mRNAs using the 2^−ΔΔCt^ method. Real-time quantitative PCR (RT-qPCR) was performed on a 7500 Thermo cycler.

### Immunofluorescence staining

CMs were grown on coverslips and then LPS (10 µg/mL) treated for 0, 15 min, 30 min, 1 h, and 2 h. After PBS washing steps, CMs were fixed with 4% paraformaldehyde, blocked with 5% BSA. Intracellular *G3BP1* and TIA-1 were detected using a rabbit-source *G3BP1* primary antibody and a mouse-source TIA-1 primary antibody, followed by incubation with a goat anti-rabbit secondary antibody conjugated with Alexa Fluor 488 and the anti-mouse immunoglobulin (Ig)G conjugated with Alexa Fluor 594, respectively. Finally, CMs were stained with DAPI to view the nuclei using a confocal laser scanning microscope (LSM800).

### Mitochondrial membrane potential assay

A Mitochondrial Membrane Potential Assay Kit with JC-1 staining was used according to the manufacturer’s protocol. In cells with high mtΔΨ, JC-1 aggregates resulted in red fluorescence; in cells with low mtΔΨ, the JC-1 monomers resulted in green fluorescence. mtΔΨ was expressed as the ratio of red to green fluorescence intensity, as determined using a Zeiss fluorescence microscope (Carl Zeiss AG, Oberkochen, Germany). ImageJ (v1.8.0) was used to quantify the fluorescence intensity. All experiments were performed in triplicate.

### Statistical analysis

GraphPad Prism 5.0 (GraphPad software, San Diego, CA, USA) was used for statistical analyses. Data are presented as the mean ± SEM. Unpaired *t*-tests or one-way analysis of variance (ANOVA) were performed for comparisons among two or multiple groups, respectively. *P* < 0.05 was considered statistically significant.

## Results

### Challenge of cardiomyocytes with lipopolysaccharide promoted SG activation via eIF2α phosphorylation

**Fig. 1:**
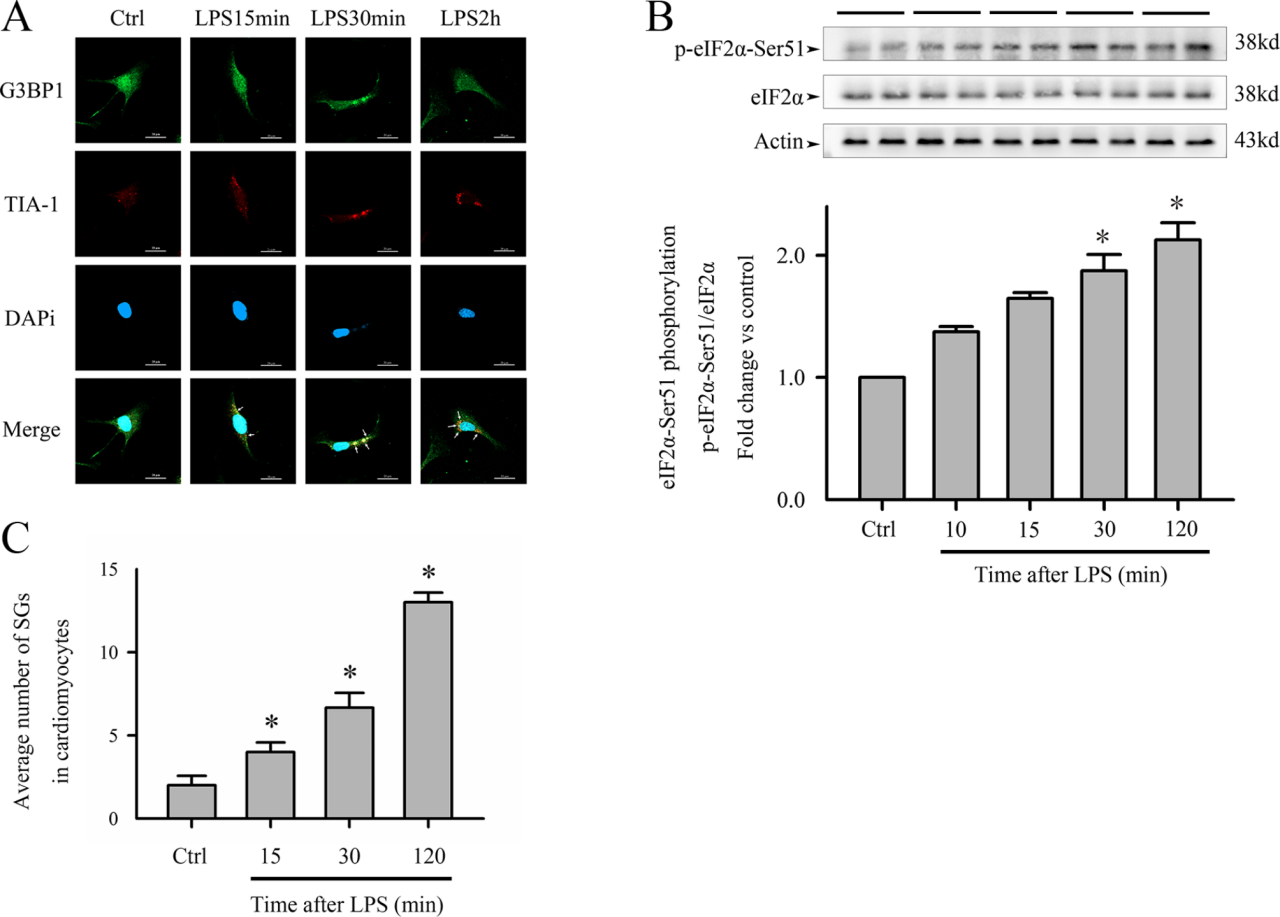
Challenge of cardiomyocytes with LPS promotes SG formation. CMs were challenged with LPS (10 µg/mL) or saline (control) for the indicated time. (**A**) Confocal microscopy analysis of SG in CMs. *G3BP1* was shown in green; TIA-1 was stained with Alexa Fluor 594 (Red), nuclei were strained with DAPI (blue). SG formation (yellow) increased after CMs were challenged with LPS. (**B**) eIF2α phosphorylation in CMs was evaluated by western blotting. Upper panel: representative blots, lower panel: densitometric analyses. (**C**) Quantification of average number of SG. n = 3 for A-C, *P < 0.05, compared with the control

CMs were treated with LPS (10 µg/mL) for 0 min, 15 min, 30 min, 1 h, and 2 h to evaluate SG activation. *G3BP1* and TIA-1 co-localization revealed by immunofluorescence staining can be used to visualize SG. The formation of SG increased remarkably after 30 min of LPS treatment and continued to increase over time (Fig. 1A). eIF2α phosphorylation levels were higher in CMs treated with LPS than in the control group (Fig. 1B), suggesting that SG formed via eIF2α phosphorylation.

### Pharmacological inhibition of SG activation aggravated lipopolysaccharide-induced myocardial inflammation and dysfunction

**Fig. 2:**
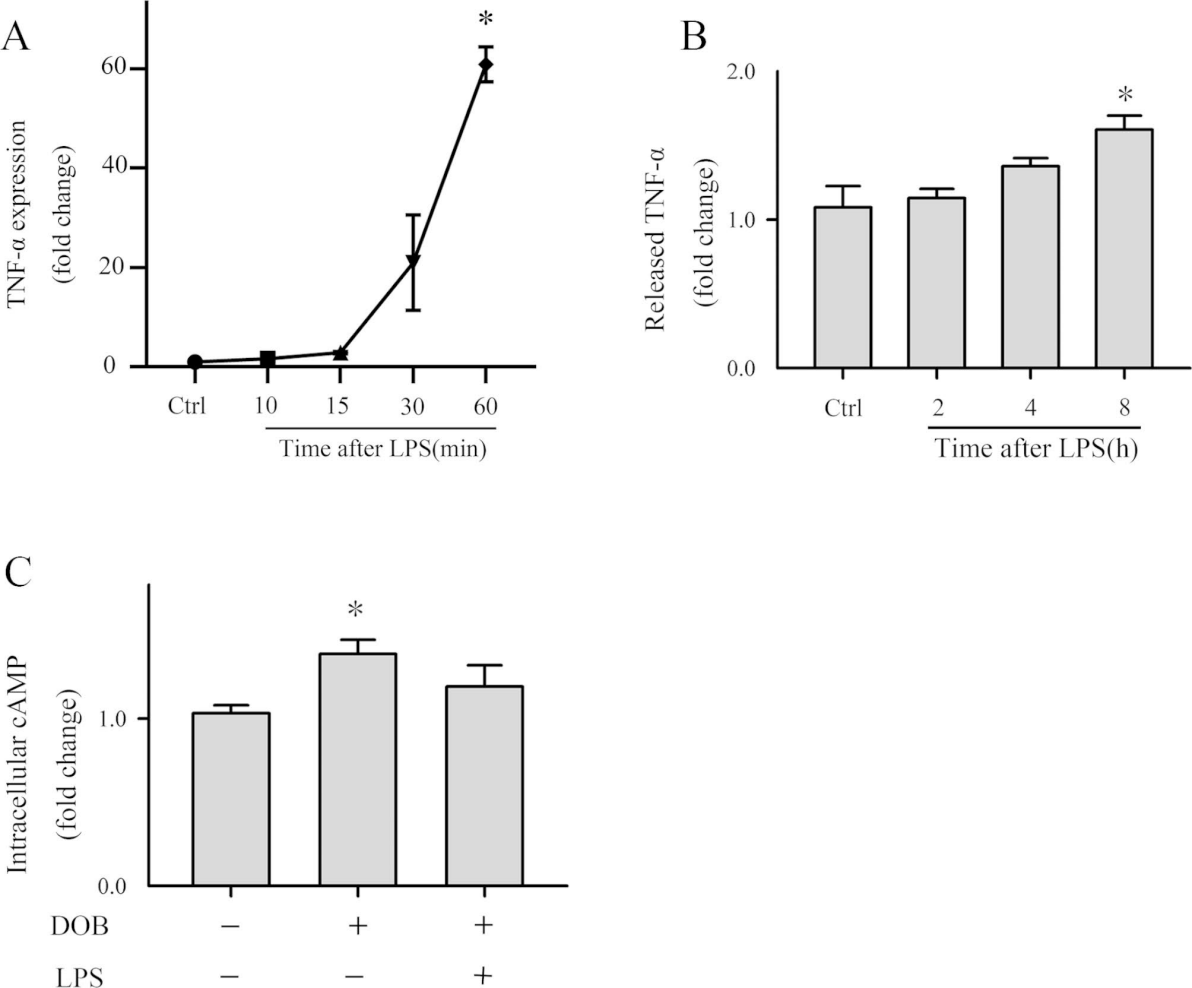
LPS increased TNF-α expression and decreased contractility in CMs. Briefly, CMs were conditioned with LPS (10 µg/mL) or saline (control), then dobutamine (10 µM) for 15 min. (**A**) *TNF-α* mRNA expression in CMs was assessed by RT-qPCR. (**B**) TNF-α protein expression was assessed by ELISA. (**C**) CMs contractility was assessed by the detection of intracellular cAMP in response to dobutamine with ELISA. n = 3, for A–C; *P < 0.05, compared with the control

To determine whether SG contributed to LPS-induced myocardial inflammation and dysfunction, the pharmacological inhibitor ISRIB was used to inhibit SG formation [24]. Previous studies have demonstrated that LPS promotes a pro-inflammatory phenotype in CMs, including increased cytokine production [25, 26]. As expected, LPS treatment increased CMs *TNF-α* mRNA levels (Fig. 2A) and TNF-α production in the supernatant (Fig. 2B). Myocyte contractile function was assessed by intracellular cAMP in response to DOB because the contractile activity of CMs is not amenable to quantitation [22]. The cAMP levels were higher in CMs challenged with DOB than in controls and decreased in LPS-treated CMs (Fig. 2C).

**Fig. 3:**
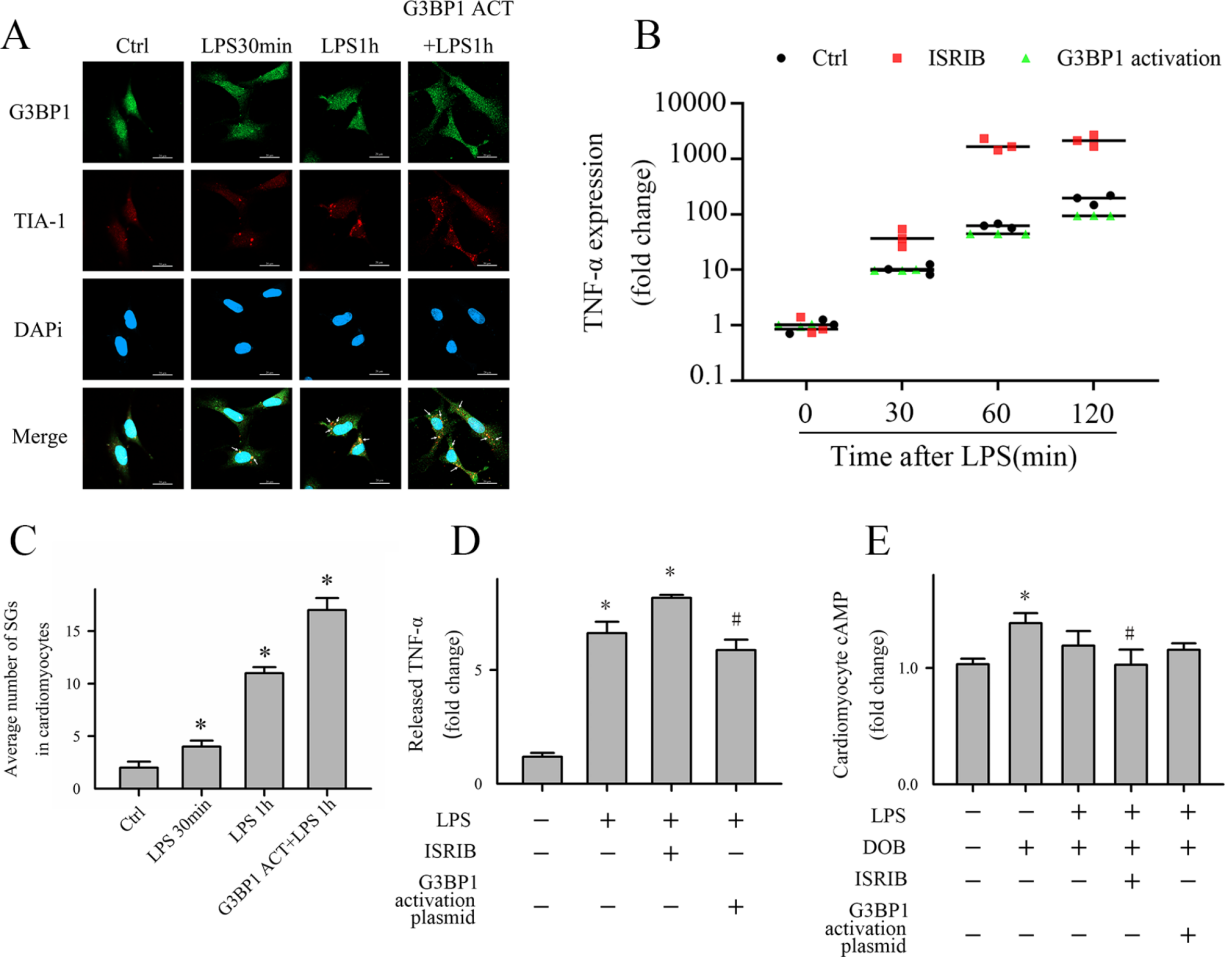
Pharmacological inhibition of SG aggravated LPS-induced TNF-α expression and the LPS-induced decrease in CMs contractility; *G3BP1* overexpression attenuated the CMs inflammatory response and dysfunction after LPS treatment. CMs were pre-treated with the SG inhibitor (ISRIB, 200 nM) or saline (control) for 30 min. Subsequently, the CMs were challenged with LPS (10 µg/mL) with or without ISRIB for the indicated time. (**A**) For *G3BP1* overexpression, CMs were transfected with the *G3BP1* CRISPR activation plasmid or with control plasmid. At 72 h after transfection, the cells were challenged with LPS (10 µg/mL) or vehicle as described above. The cells (5 × 10^5^/well) were grown on coverslips and then prepared for immunofluorescence according to the protocol described in Fig. 1A. (**B**) RT-qPCR measured *TNF-α* expression quantification. (**C**) Quantification of average number of SG. (**D**) CMs TNF-α expression was assessed by ELISA. (**E**) CMs contractility was determined by the detection of intracellular cAMP in response to dobutamine. N = 3, for all experiments. *P < 0.05 as compared with control; #*P* < 0.05 as compared with columns marked with*. ACT, activation plasmid

Afterward, CMs were treated with ISRIB and LPS. TNF-α expression increased after ISRIB treatment (Fig. 3B/D), and the difference in intracellular cAMP levels between the LPS and control samples increased (Fig. 3E). These data showed that ISRIB negatively affected the cAMP response to DOB and exacerbated myocardial inflammation, indicating that SG formation plays a protective role in sepsis, preventing SIMD.

### *G3BP1* overexpression increased SG formation and attenuated lipopolysaccharide-induced myocardial inflammation and dysfunction

To further elucidate the effect of SG activation on myocardial dysfunction in sepsis, we overexpressed *G3BP1* to promote SG formation [27] in CMs with a CRISPR-Cas9 plasmid. *G3BP1* overexpression increased SG formation (Fig. 3A/C). In addition, the increase in SG attenuated the increase in CMs TNF-α expression, and CMs contractility is also improved, as indicated by increased levels of intracellular cAMP in CMs treated with LPS (Fig. 3B/D/E).

### SG prevented sepsis-induced mitochondrial membrane potential dissipation

**Fig. 4:**
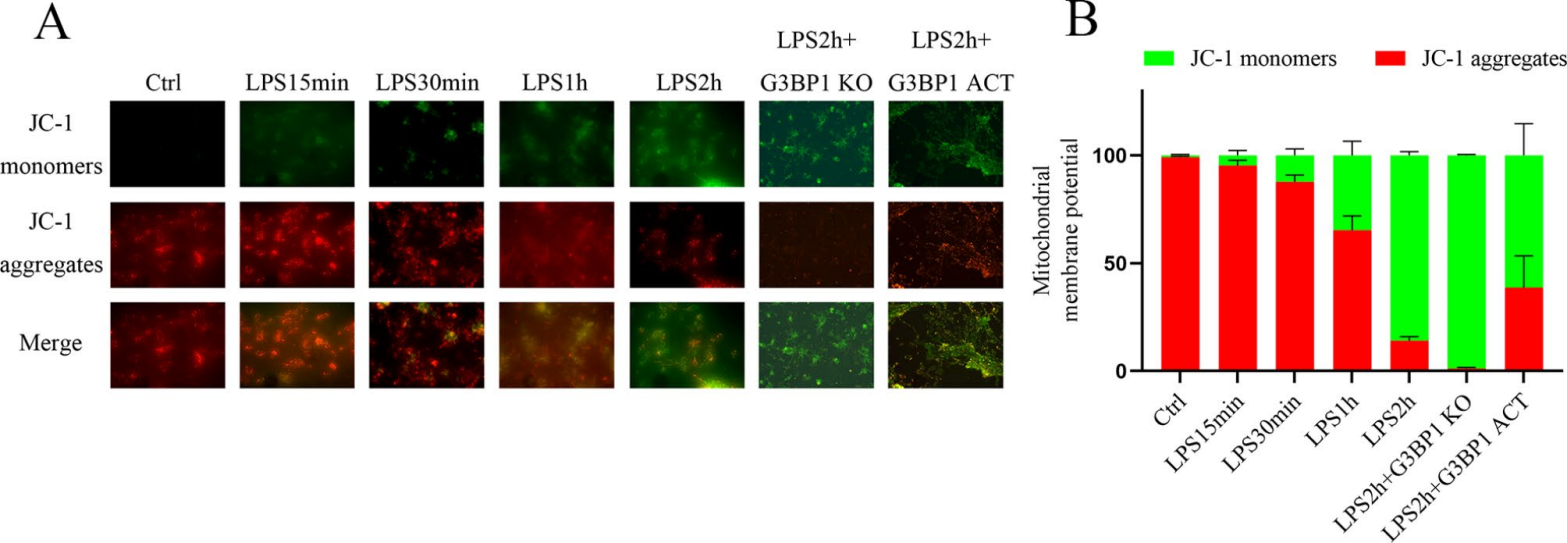
Increase in SG formation attenuates mitochondrial membrane potential depolarization in CMs with LPS. CMs were treated with LPS (10 µg/mL) for different times, as indicated in the figure, while two other sets of CMs were transfected with the *G3BP1* KO plasmid or *G3BP1* activation plasmid with LPS for 2 h. Subsequently, mitochondrial membrane potential was assessed using a JC‑1 probe by fluorescence microscopy. Mitochondrial membrane depolarization is indicated by a shift from red to green fluorescence. (**A**) Representative images. (**B**) Quantification results are presented. Bar graphs represent the mean ± SEM; n = 3 for A and B, KO, knockout plasmid; SEM, standard error of the mean

As SG exert a cardioprotective role, we studied their effect on mitochondrial membrane potential using JC-1 [28]. The challenge of CMs with LPS depolarized the mitochondrial membrane; *G3BP1* CRISPR KO plasmid exacerbated membrane depolarization and the *G3BP1* activation plasmid had the opposite effect (Fig. 4A–B).

## Discussion

The results of this study showed that LPS stimulation activates SG in CMs via the classical eIF2α phosphorylation pathway. The inhibition of SG formation promoted LPS-induced myocardial injury, while increased SG formation attenuated LPS-induced CMs dysfunction. These findings clearly demonstrated that SG exert a protective effect against CM dysfunction in sepsis.

SG are involved in the regulation of various cell signaling pathways and are associated with many diseases. SG activation indicates a “state of emergency” by blocking or amplifying key components of those pathways [11, 29]. Even without membranes, SG have basic functions as an intracellular chamber, as well as concentration, storage, isolation, and signal transduction. When specific proteins are sequestered in the SG, their confinement in a negative orientation has cytoprotective effects and vice versa [30]. For example, when the dead nuclear protein DDX3X localizes to SG, nuclear factor kappa-B (NF-κB)-dependent pro-inflammatory and pro-apoptotic responses are inhibited, which prevents apoptosis [31]. SG formation occurs prior to the intracellular inflammatory response and apoptosis, suggesting that SG act as an upstream signaling hub by recruiting core signal proteins and can determine the responses of cells to stress [11]. The inhibition of *G3BP1* by siRNA gene silencing significantly affects the expression of inflammation-related genes [32]. We demonstrated that *G3BP1* overexpression mediated the quick formation of SG in CMs (Fig. 3A), suggesting that SG affect the LPS-induced inflammatory response and CMs contractility and supporting the key role of *G3BP1* in the core SG network.

Even though the pathogenesis of septic cardiomyopathy is not well understood, septic myocardial damage rarely occurs at the organ level. Many studies have shown that SIMD is characterized by a lack of significant cell death, normal tissue oxygen levels, and myocardial dysfunction reversal in survivors [33, 34]. Mitochondrial activity is closely related to myocardial function [35]. In this study, we prove evidence for the correlation between SG and mitochondrial function in SIMD, suggesting that SG activation is like a brake, acting as a buffer against the septic inflammatory response and mitochondrial dysfunction. In addition, SG dynamics require ATP, which promotes reduced metabolic activity to adapt to insufficient ATP production caused by mitochondrial dysfunction [36]. Since the regulatory mechanism of septic cardiomyopathy mostly occurs at the cellular level, is it possible that the dynamic activation and disassembly of SG are related to the reversible mechanism underlying myocardial injury in sepsis [37]. Although this hypothesis has not been proven, it is worthy of further research.

SG are highly conserved in eukaryotes and are closely related to other cardiovascular diseases, such as atrial fibrillation and heart failure, where mitochondrial dysfunction and inflammation also play important roles [38, 39].SG are rapidly induced in atrial fibrillation and confer cytoprotection against oxidative stress, calcium overload, and atrial fibrosis [39]. SG have been detected both in vivo and in vitro and play an active role in vascular smooth muscle cells and macrophages during atherosclerosis [20]. Inflammatory activation in diabetic cardiomyopathy is accompanied by a reduction in SG and worsened clinical prognosis [38]. The presence of SG has also been observed in animal models of heart failure [40]. These observations are consistent with the protective role of SG and suggest that SG could be therapeutic target for SIMD.

However, the beneficial effects of SG are still debatable [29, 30]. It is possible that SG activation initially helps cells survive under stress, while long-term SG may be a by-product of this regulation and promote various diseases [41]. SG have complex and dynamic functions and are difficult to purify in vitro, making their clinical application quite challenging [10, 42, 43]. Here, we focused on the role of SG activation in SIMD without considering the impact of SG depolymerization or protein–protein interactions that occur in SG [44]. Characterizing the differences in SG composition and protein dynamics may be a key to understanding the mechanism underlying SG-related diseases in the future.

Our results identify a protective role of SG activation for CMs in LPS-induced myocardial dysfunction. However, the use of a cell-based model is a key limitation of this work [45]. Moreover, humans are more sensitive to LPS than rodents [46]. Therefore, myocytes grown in culture may not fully represent those in whole animal systems; thus, the role of SG activation in the clinical setting needs to be explored further. In summary, caution should be applied in extrapolating these results.

## Conclusions

SG in CMs exert a protective effect against LPS-induced myocardial dysfunction, suggesting that they are a potential therapeutic target for SIMD.

## Electronic supplementary material

Below is the link to the electronic supplementary material.

Supplementary Material 1

## Data Availability

The datasets used and/or analyzed during the current study available from the corresponding author on reasonable request.

## Abbreviations

cAMP: cyclic adenosine monophosphate
CM: cardiomyocytes
eIF2α: eukaryotic translation initiation factor alpha
G3BP1: RAS-GAP SH3 binding protein 1
LPS: lipopolysaccharide
NF-κB: nuclear factor kappa-B
SG: stress granules
SIMD: sepsis-induced myocardial dysfunction
TIA-1: T cell-restricted intracellular antigen 1
TNF-α: tumor necrosis factor alpha
