# Supplementary figures and images for: Stress granule activation attenuates lipopolysaccharide-induced cardiomyocyte dysfunction
Source: BMC Cardiovasc Disord. 2023 May 27;23:277. doi: 10.1186/s12872-023-03281-0 (PMC10265778; doi:10.1186/s12872-023-03281-0)

P-eIF2 $\alpha$

eIF2 $\alpha$

## Actin

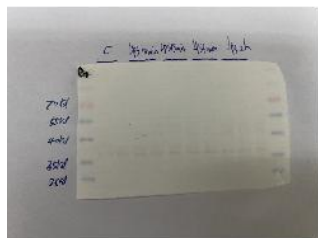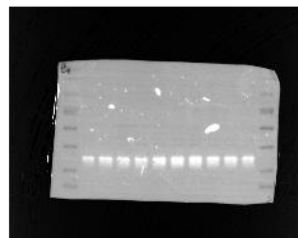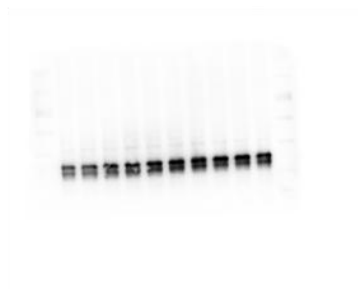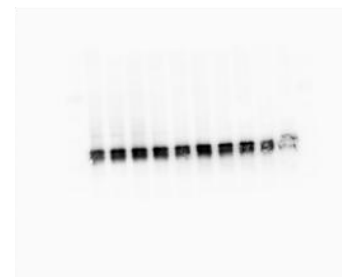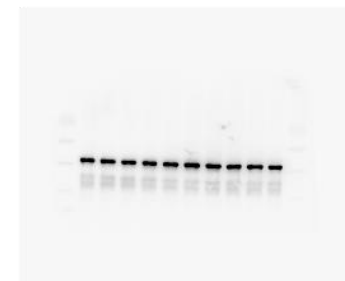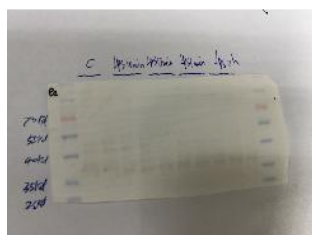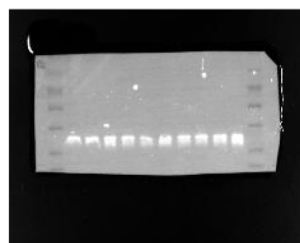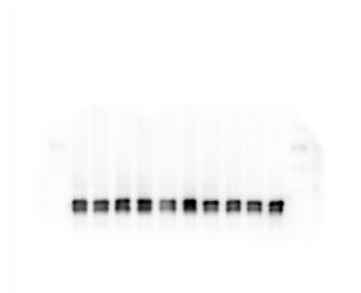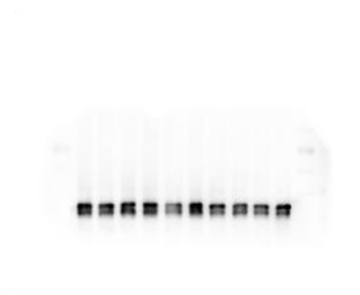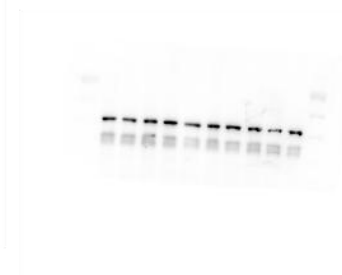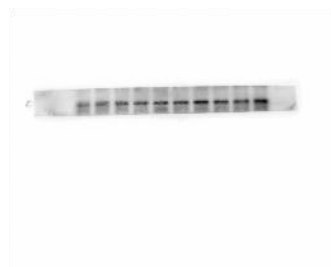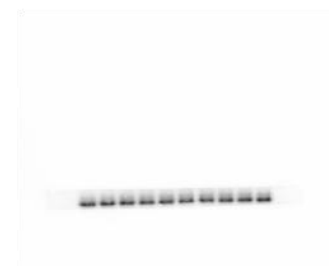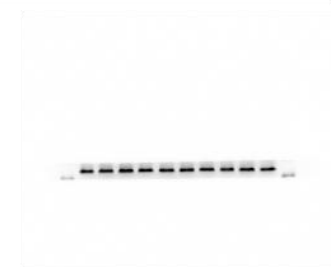

Supplement: Supplementary file 1 — Supplementary Material 1 [file 12872_2023_3281_MOESM1_ESM.pdf]
